# (*Z*)-1-(2,4-Difluoro­phen­yl)-2-(1*H*-1,2,4-triazol-1-yl)ethanone oxime

**DOI:** 10.1107/S1600536811050008

**Published:** 2011-11-30

**Authors:** Guang-yan Yu, Chen Li, Tao Xiao, Song Li, Xin Tian

**Affiliations:** a Department of Applied Chemistry, College of Science, Nanjing University of Technology, Nanjing 210009, People’s Republic of China

## Abstract

In the title compound, C_10_H_8_F_2_N_4_O, the dihedral angle between the rings is 65.4 (1)°. In the crystal, inter­molecular O—H⋯N and C—H⋯F hydrogen bonds link the mol­ecules in a stacked arrangement along the *a* and *c* axes, respectively.

## Related literature

For applications of related compounds, see: Foroumadi *et al.* (2003 ▶); Mixich & Thiele (1979 ▶); Wolfgang *et al.* (1981 ▶). For a related structure, see: Tao *et al.* (2007 ▶). For standard bond lengths, see: Allen *et al.* (1987 ▶).
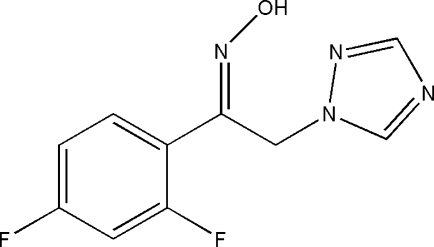


## Experimental

### 

#### Crystal data

C_10_H_8_F_2_N_4_O
*M* *_r_* = 238.20
Monoclinic, 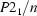

*a* = 8.6320 (17) Å
*b* = 12.433 (3) Å
*c* = 10.417 (2) Å
β = 104.85 (3)°
*V* = 1080.6 (4) Å^3^
*Z* = 4
Mo *K*α radiation
μ = 0.12 mm^−1^
*T* = 293 K
0.30 × 0.20 × 0.10 mm

#### Data collection

Enraf–Nonius CAD-4 diffractometer
Absorption correction: ψ scan (North *et al.*, 1968 ▶) *T*_min_ = 0.964, *T*_max_ = 0.988
3127 measured reflections
1987 independent reflections
1217 reflections with *I* > 2σ(*I*)
*R*_int_ = 0.042
3 standard reflections every 200 reflections  intensity decay: 1%

#### Refinement

*R*[*F* ^2^ > 2σ(*F* ^2^)] = 0.060
*wR*(*F* ^2^) = 0.171
*S* = 1.01
1987 reflections
154 parameters
H-atom parameters constrained
Δρ_max_ = 0.53 e Å^−3^
Δρ_min_ = −0.22 e Å^−3^

### 

Data collection: *CAD-4 EXPRESS* (Enraf–Nonius, 1994 ▶); cell refinement: *CAD-4 EXPRESS*; data reduction: *XCAD4* (Harms & Wocadlo, 1995 ▶); program(s) used to solve structure: *SHELXS97* (Sheldrick, 2008 ▶); program(s) used to refine structure: *SHELXL97* (Sheldrick, 2008 ▶); molecular graphics: *PLATON* (Spek, 2009 ▶); software used to prepare material for publication: *SHELXTL* (Sheldrick, 2008 ▶).

## Supplementary Material

Crystal structure: contains datablock(s) I, global. DOI: 10.1107/S1600536811050008/zq2136sup1.cif

Structure factors: contains datablock(s) I. DOI: 10.1107/S1600536811050008/zq2136Isup2.hkl

Supplementary material file. DOI: 10.1107/S1600536811050008/zq2136Isup3.cml

Additional supplementary materials:  crystallographic information; 3D view; checkCIF report

## Figures and Tables

**Table 1:** Hydrogen-bond geometry (Å, °)

*D*—H⋯*A*	*D*—H	H⋯*A*	*D*⋯*A*	*D*—H⋯*A*
O1—H1*A*⋯N4^i^	0.82	1.94	2.764 (3)	176
C10—H10⋯F1^ii^	0.93	2.47	3.289 (4)	148

Symmetry codes: (i) 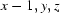; (ii) 
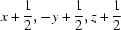
.

**Fig. 1.**
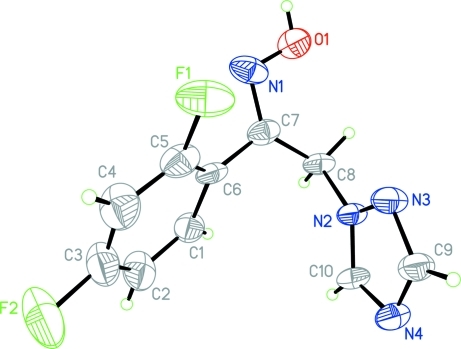
The molecular structure of the title compound showing displacement ellipsoids drawn at the 50% probability level.

**Fig. 2.**
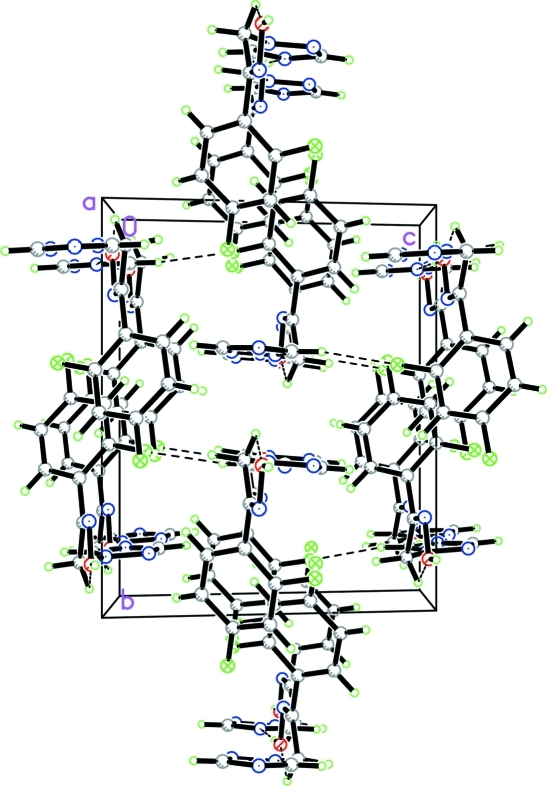
A packing diagram of the title compound. Hydrogen bonds are shown as dashed lines.

## supplementary crystallographic information

### Comment

The title compound, C_10_H_8_O_1_N_4_F_2_, is the key intermediate in the synthesis of a new kind of antifungal drug (Tao *et al.*, 2007; Foroumadi *et al.*, 2003; Wolfgang *et al.*, 1981) and exhibits a chemical structure similar to oxiconazole (Mixich & Thiele, 1979). We designed and synthesized the title compound, and we herein report its crystal structure (Fig. 1).

The bond lengths are within normal ranges (Allen *et al.*, 1987). The dihedral angle between rings A (N2-N4/C9/C10) and B (C1-C6) is 65.4 (1) °. In the crystal structure, intermolecular intermolecular O–H···N and C-H···F hydrogen bonds (Table 2) link the molecules in a stacked arrangement along along the *a* and *c* axes, respectively (Fig. 2).

### Experimental

Hydroxylammonium chloride (3 g, 43.2 mmol) dissolved in ethanol (20 ml) was dropwise added to a solution of 1-(2,4-difluorophenyl)-2-(1*H*-1,2,4-triazol-1-yl)ethanone (5 g,22.4 mmol) in ethanol (50 ml) which contained CH_3_COONa (4 g, 48.8 mmol) under reflux conditions for 4 h. The mixture was placed in ice-water (100 ml), and the crystalline product was isolated by filtration, washed with water (100 ml). The crystals were obtained by dissolving the product in ethanol (20 ml) and evaporating acetone slowly at room temperature for about 7 d.

### Refinement

The H atom of the hydroxy group was located in a Fourier difference map but was constrained to ride on the parent atom with O—H = 0.82 Å and *U*_iso_(H) = 1.5*U*_eq_(O). The other H atoms were positioned geometrically with C—H = 0.93 Å (aromatic) and 0.97 Å (methylene) and constrained to ride on their parent atoms with *U*_iso_(H) = 1.2*U*_eq_(C).

### Crystal data

**Table:**

C_10_H_8_F_2_N_4_O	*F*(000) = 488
*M**_r_* = 238.20	*D*_x_ = 1.464 Mg m^−^^3^
Monoclinic, *P*2_1_/*n*	Melting point: 400 K
Hall symbol: -P 2yn	Mo *K*α radiation, λ = 0.71073 Å
*a* = 8.6320 (17) Å	Cell parameters from 25 reflections
*b* = 12.433 (3) Å	θ = 9–13°
*c* = 10.417 (2) Å	µ = 0.12 mm^−^^1^
β = 104.85 (3)°	*T* = 293 K
*V* = 1080.6 (4) Å^3^	Black, white
*Z* = 4	0.30 × 0.20 × 0.10 mm

### Data collection

**Table:**

Enraf–Nonius CAD-4 diffractometer	1217 reflections with *I* > 2σ(*I*)
Radiation source: fine-focus sealed tube	*R*_int_ = 0.042
graphite	θ_max_ = 25.4°, θ_min_ = 2.6°
ω/2θ scans	*h* = 0→10
Absorption correction: ψ scan (North *et al.*, 1968)	*k* = −5→14
*T*_min_ = 0.964, *T*_max_ = 0.988	*l* = −12→12
3127 measured reflections	3 standard reflections every 200 reflections
1987 independent reflections	intensity decay: 1%

### Refinement

**Table:**

Refinement on *F*^2^	Secondary atom site location: difference Fourier map
Least-squares matrix: full	Hydrogen site location: inferred from neighbouring sites
*R*[*F*^2^ > 2σ(*F*^2^)] = 0.060	H-atom parameters constrained
*wR*(*F*^2^) = 0.171	*w* = 1/[σ^2^(*F*_o_^2^) + (0.090*P*)^2^] where *P* = (*F*_o_^2^ + 2*F*_c_^2^)/3
*S* = 1.01	(Δ/σ)_max_ < 0.001
1987 reflections	Δρ_max_ = 0.53 e Å^−^^3^
154 parameters	Δρ_min_ = −0.22 e Å^−^^3^
0 restraints	Extinction correction: *SHELXL97* (Sheldrick, 2008)
Primary atom site location: structure-invariant direct methods	Extinction coefficient: 0.034 (5)

### Special details

**Table:**

Geometry. All e.s.d.'s (except the e.s.d. in the dihedral angle between two l.s. planes) are estimated using the full covariance matrix. The cell e.s.d.'s are taken into account individually in the estimation of e.s.d.'s in distances, angles and torsion angles; correlations between e.s.d.'s in cell parameters are only used when they are defined by crystal symmetry. An approximate (isotropic) treatment of cell e.s.d.'s is used for estimating e.s.d.'s involving l.s. planes.
Refinement. Refinement of *F*^2^ against ALL reflections. The weighted *R*-factor *wR* and goodness of fit *S* are based on *F*^2^, conventional *R*-factors *R* are based on *F*, with *F* set to zero for negative *F*^2^. The threshold expression of *F*^2^ > σ(*F*^2^) is used only for calculating *R*-factors(gt) *etc*. and is not relevant to the choice of reflections for refinement. *R*-factors based on *F*^2^ are statistically about twice as large as those based on *F*, and *R*- factors based on ALL data will be even larger.

### Fractional atomic coordinates and isotropic or equivalent isotropic displacement parameters (Å^2^)

**Table:**

	*x*	*y*	*z*	*U*_iso_*/*U*_eq_	
O1	−0.0689 (3)	0.1304 (2)	0.0266 (2)	0.0885 (7)	
H1A	−0.1670	0.1325	0.0102	0.133*	
F1	0.0628 (2)	0.39466 (19)	−0.12253 (18)	0.1082 (8)	
N1	−0.0109 (3)	0.2303 (3)	0.0363 (2)	0.0787 (8)	
C1	0.3526 (3)	0.3487 (3)	0.1896 (3)	0.0689 (8)	
H1	0.3862	0.2944	0.2520	0.083*	
N2	0.3725 (2)	0.13025 (17)	0.0097 (2)	0.0535 (6)	
C2	0.4296 (4)	0.4452 (3)	0.2083 (3)	0.0880 (11)	
H2	0.5142	0.4572	0.2826	0.106*	
F2	0.4618 (4)	0.61943 (18)	0.1316 (3)	0.1508 (12)	
N3	0.3361 (2)	0.1384 (2)	−0.1226 (2)	0.0654 (7)	
C3	0.3801 (5)	0.5240 (3)	0.1156 (4)	0.0917 (10)	
N4	0.6005 (2)	0.1436 (2)	−0.0387 (2)	0.0679 (7)	
C4	0.2564 (4)	0.5106 (3)	0.0065 (3)	0.0944 (11)	
H4	0.2229	0.5659	−0.0545	0.113*	
C5	0.1822 (3)	0.4114 (3)	−0.0101 (3)	0.0707 (9)	
C6	0.2250 (3)	0.3288 (2)	0.0798 (2)	0.0538 (7)	
C7	0.1461 (3)	0.2225 (3)	0.0625 (2)	0.0619 (8)	
C8	0.2467 (3)	0.1224 (2)	0.0803 (3)	0.0605 (8)	
H8A	0.2951	0.1110	0.1741	0.073*	
H8B	0.1789	0.0609	0.0471	0.073*	
C9	0.4781 (3)	0.1454 (2)	−0.1470 (3)	0.0663 (8)	
H9	0.4918	0.1511	−0.2324	0.080*	
C10	0.5286 (3)	0.1362 (2)	0.0578 (3)	0.0597 (7)	
H10	0.5810	0.1353	0.1477	0.072*	

### Atomic displacement parameters (Å^2^)

**Table:**

	*U*^11^	*U*^22^	*U*^33^	*U*^12^	*U*^13^	*U*^23^
O1	0.0712 (14)	0.0982 (19)	0.0951 (17)	0.0097 (14)	0.0198 (12)	−0.0037 (14)
F1	0.0744 (12)	0.160 (2)	0.0717 (12)	−0.0057 (12)	−0.0146 (10)	0.0297 (11)
N1	0.0610 (14)	0.115 (3)	0.0608 (15)	−0.0278 (15)	0.0177 (11)	−0.0027 (15)
C1	0.0595 (16)	0.078 (2)	0.0599 (16)	0.0107 (16)	−0.0024 (13)	−0.0075 (15)
N2	0.0337 (10)	0.0662 (15)	0.0611 (13)	−0.0045 (10)	0.0133 (9)	0.0006 (11)
C2	0.095 (2)	0.087 (3)	0.0639 (19)	0.012 (2)	−0.0120 (18)	−0.0177 (19)
F2	0.183 (3)	0.0744 (16)	0.161 (2)	−0.0211 (16)	−0.017 (2)	−0.0079 (15)
N3	0.0441 (11)	0.093 (2)	0.0577 (13)	−0.0116 (12)	0.0107 (10)	−0.0005 (12)
C3	0.106 (3)	0.067 (2)	0.091 (2)	0.002 (2)	0.004 (2)	−0.014 (2)
N4	0.0449 (12)	0.0795 (18)	0.0822 (16)	−0.0011 (11)	0.0216 (12)	−0.0008 (13)
C4	0.108 (3)	0.078 (3)	0.085 (2)	0.016 (2)	0.002 (2)	0.014 (2)
C5	0.0594 (16)	0.100 (3)	0.0456 (15)	0.0192 (18)	0.0007 (13)	0.0074 (16)
C6	0.0283 (11)	0.084 (2)	0.0503 (14)	0.0060 (12)	0.0120 (10)	−0.0023 (14)
C7	0.0463 (13)	0.096 (2)	0.0464 (14)	0.0039 (15)	0.0175 (11)	0.0027 (14)
C8	0.0363 (12)	0.080 (2)	0.0674 (17)	−0.0076 (13)	0.0175 (12)	0.0078 (15)
C9	0.0470 (14)	0.089 (2)	0.0659 (17)	−0.0109 (15)	0.0205 (13)	−0.0046 (16)
C10	0.0297 (11)	0.075 (2)	0.0724 (17)	0.0046 (12)	0.0091 (11)	0.0088 (14)

### Geometric parameters (Å, °)

**Table:**

O1—N1	1.334 (3)	N3—C9	1.317 (3)
O1—H1A	0.8200	C3—C4	1.356 (5)
F1—C5	1.364 (3)	N4—C10	1.313 (3)
N1—C7	1.315 (3)	N4—C9	1.333 (3)
C1—C2	1.362 (4)	C4—C5	1.380 (5)
C1—C6	1.392 (3)	C4—H4	0.9300
C1—H1	0.9300	C5—C6	1.375 (4)
N2—C10	1.314 (3)	C6—C7	1.477 (4)
N2—N3	1.337 (3)	C7—C8	1.502 (4)
N2—C8	1.463 (3)	C8—H8A	0.9700
C2—C3	1.365 (5)	C8—H8B	0.9700
C2—H2	0.9300	C9—H9	0.9300
F2—C3	1.368 (4)	C10—H10	0.9300
			
N1—O1—H1A	109.5	F1—C5—C4	117.9 (3)
C7—N1—O1	107.1 (3)	C6—C5—C4	123.2 (3)
C2—C1—C6	121.9 (3)	C5—C6—C1	116.4 (3)
C2—C1—H1	119.0	C5—C6—C7	123.4 (2)
C6—C1—H1	119.0	C1—C6—C7	120.1 (3)
C10—N2—N3	109.6 (2)	N1—C7—C6	112.2 (3)
C10—N2—C8	129.3 (2)	N1—C7—C8	128.2 (3)
N3—N2—C8	120.97 (18)	C6—C7—C8	119.5 (2)
C1—C2—C3	118.6 (3)	N2—C8—C7	111.3 (2)
C1—C2—H2	120.7	N2—C8—H8A	109.4
C3—C2—H2	120.7	C7—C8—H8A	109.4
C9—N3—N2	102.7 (2)	N2—C8—H8B	109.4
C4—C3—C2	122.8 (4)	C7—C8—H8B	109.4
C4—C3—F2	118.6 (4)	H8A—C8—H8B	108.0
C2—C3—F2	118.6 (3)	N3—C9—N4	114.2 (2)
C10—N4—C9	102.8 (2)	N3—C9—H9	122.9
C3—C4—C5	117.1 (3)	N4—C9—H9	122.9
C3—C4—H4	121.5	N4—C10—N2	110.6 (2)
C5—C4—H4	121.5	N4—C10—H10	124.7
F1—C5—C6	118.8 (3)	N2—C10—H10	124.7
			
C6—C1—C2—C3	−0.5 (5)	O1—N1—C7—C6	−178.2 (2)
C10—N2—N3—C9	2.1 (3)	O1—N1—C7—C8	−0.5 (4)
C8—N2—N3—C9	179.0 (2)	C5—C6—C7—N1	−50.9 (3)
C1—C2—C3—C4	0.7 (6)	C1—C6—C7—N1	130.8 (3)
C1—C2—C3—F2	−177.3 (3)	C5—C6—C7—C8	131.1 (3)
C2—C3—C4—C5	−1.5 (6)	C1—C6—C7—C8	−47.1 (3)
F2—C3—C4—C5	176.6 (3)	C10—N2—C8—C7	112.4 (3)
C3—C4—C5—F1	−176.8 (3)	N3—N2—C8—C7	−63.8 (3)
C3—C4—C5—C6	2.0 (5)	N1—C7—C8—N2	135.8 (3)
F1—C5—C6—C1	177.1 (2)	C6—C7—C8—N2	−46.7 (3)
C4—C5—C6—C1	−1.7 (4)	N2—N3—C9—N4	−0.9 (3)
F1—C5—C6—C7	−1.2 (4)	C10—N4—C9—N3	−0.7 (4)
C4—C5—C6—C7	180.0 (3)	C9—N4—C10—N2	2.0 (3)
C2—C1—C6—C5	0.9 (4)	N3—N2—C10—N4	−2.7 (3)
C2—C1—C6—C7	179.3 (3)	C8—N2—C10—N4	−179.2 (3)

### Hydrogen-bond geometry (Å, °)

**Table:**

*D*—H···*A*	*D*—H	H···*A*	*D*···*A*	*D*—H···*A*
O1—H1A···N4^i^	0.82	1.94	2.764 (3)	176
C10—H10···F1^ii^	0.93	2.47	3.289 (4)	148

Symmetry codes: (i) *x*−1, *y*, *z*; (ii) *x*+1/2, −*y*+1/2, *z*+1/2.
